# Genomic Landscape of Myelodysplastic/Myeloproliferative Neoplasms: A Multi-Central Study

**DOI:** 10.3390/ijms251810214

**Published:** 2024-09-23

**Authors:** Fei Fei, Amar Jariwala, Sheeja Pullarkat, Eric Loo, Yan Liu, Parastou Tizro, Haris Ali, Salman Otoukesh, Idoroenyi Amanam, Andrew Artz, Feras Ally, Milhan Telatar, Ryotaro Nakamura, Guido Marcucci, Michelle Afkhami

**Affiliations:** 1 Department of Pathology, City of Hope Comprehensive Cancer Center, Duarte, CA 91010, USA; ffei@coh.org (F.F.); amarjari@gwmail.gwu.edu (A.J.);; 2 Fulgent Oncology, 4399 Santa Anita Ave, El Monte, CA 91731, USA; 3 Department of Pathology, Division of Hematopathology, University of Los Angeles Medical Center, Los Angeles, CA 90095, USA; 4 Department of Pathology, Dartmouth–Hitchcock Medical Center, Lebanon, NH 03756, USA; 5 Department of Pathology and Laboratory Medicine, Loma Linda University Health, Loma Linda, CA 92350, USA; 6 Department of Hematology and Hematopoietic Cell Transplantation, City of Hope Comprehensive Cancer Center, Duarte, CA 91010, USA; 7 Department of Pathology, University of Washington Medical Center, Seattle, WA 98195, USA

**Keywords:** myelodysplastic syndrome/myeloproliferative neoplasm (MDS/MPN), next-generation sequencing, genomic, myeloid neoplasm, gene mutation

## Abstract

The accurate diagnosis and classification of myelodysplastic/myeloproliferative neoplasm (MDS/MPN) are challenging due to the overlapping pathological and molecular features of myelodysplastic syndrome (MDS) and myeloproliferative neoplasm (MPN). We investigated the genomic landscape in different MDS/MPN subtypes, including chronic myelomonocytic leukemia (CMML; n = 97), atypical chronic myeloid leukemia (aCML; n = 8), MDS/MPN-unclassified (MDS/MPN-U; n = 44), and MDS/MPN with ring sideroblasts and thrombocytosis (MDS/MPN-RS-T; n = 12). Our study indicated that MDS/MPN is characterized by mutations commonly identified in myeloid neoplasms, with *TET2* (52%) being the most frequently mutated gene, followed by *ASXL1* (38.7%), *SRSF2* (34.7%), and *JAK2* (19.7%), among others. However, the distribution of recurrent mutations differs across the MDS/MPN subtypes. We confirmed that specific gene combinations correlate with specific MDS/MPN subtypes (e.g., *TET2/SRSF2* in CMML, *ASXL1/SETBP1* in aCML, and *SF3B1/JAK2* in MDS/MPN-RS-T), with MDS/MPN-U being the most heterogeneous. Furthermore, we found that older age (≥65 years) and mutations in *RUNX1* and *TP53* were associated with poorer clinical outcomes in CMML (*p* < 0.05) by multivariate analysis. In MDS/MPN-U, *CBL* mutations (*p* < 0.05) were the sole negative prognostic factors identified in our study by multivariate analysis (*p* < 0.05). Overall, our study provides genetic insights into various MDS/MPN subtypes, which may aid in diagnosis and clinical decision-making for patients with MDS/MPN.

## 1. Introduction

Myelodysplastic/myeloproliferative neoplasm (MDS/MPN) is a category of myeloid neoplasms characterized by overlapping pathological and molecular features of myelodysplastic syndrome (MDS) and myeloproliferative neoplasm (MPN), often manifesting clinically with various combinations of cytopenias and cytoses [1]. According to the 2017 WHO Classification of Tumours of Haematopoietic and Lymphoid Tissues, this category includes chronic myelomonocytic leukemia (CMML), atypical chronic myeloid leukemia, *BCR-ABL1* negative (aCML), myelodysplastic/myeloproliferative neoplasm with ring sideroblasts and thrombocytosis (MDS/MPN-RS-T), myelodysplastic/myeloproliferative neoplasm, unclassifiable (MDS/MPN-U), and juvenile myelomonocytic leukemia (JMML) [2].

Identifying a clonal abnormality, especially when dysplasia is minimal or absent, plays an important role in the diagnostic criteria for MDS/MPN. However, recurrent cytogenetic abnormalities are only noted in approximately 30–40% of MDS/MPN cases [3,4]. Thus, diagnosis and classification can be very challenging with normal cytogenetics and borderline morphologic findings, particularly in the presence of confounding factors, such as medication effects, toxins, infections, and autoimmune diseases [4]. Additionally, CMML, aCML, and MDS/MPN-U have been suggested to represent a continuum of related diseases rather than discrete diagnostic entities, as indicated by whole-exome and RNA sequencing. This, in part, explains the difficulty in making clear diagnostic classifications based solely on morphological findings in certain cases [5]. Recently, the 2022 fifth edition of the WHO Classification of Tumours of Haematopoietic and Lymphoid Tissues [1] emphasizes the integration of comprehensive genetic testing into the diagnosis, prognosis, and treatment of patients with MDS/MPN. Previous studies have found that although cytogenetic abnormalities and somatic copy number variations are uncommon, more than 90% of patients with MDS/MPN harbor somatic mutations in myeloid-related genes, none of which are specific to MDS/MPN [6].

Significant efforts have been made to understand how molecular signatures impact pathogenesis and disease evolution in MDS/MPN, particularly in CMML. Previous studies have shown that *TET2*, *ASXL1*, and *SRSF2* mutations are commonly associated with CMML and that certain mutation patterns can be predictive of prognosis. *ASXL1* mutations are known to be associated with an unfavorable prognosis, while *TET2* mutations have been linked to favorable clinical outcomes in CMML [7,8]. Cargo et al.’s study demonstrates that the presence of certain mutations (*ASXL1*, *CBL*, *DNMT3A*, *NRAS*, and *RUNX1*) correlates with similar immunophenotypes and overall survival (OS), regardless of whether the patient was diagnosed with CMML [9]. Additionally, *ASXL1* and/or *NRAS* mutations may impact allogeneic hematopoietic stem cell transplantation (HSCT) outcomes in CMML [10]. However, the molecular features in other subtypes of MDS/MPN have not been widely investigated. aCML, known as MDS/MPN with neutrophilia, according to the recent fifth edition of the WHO classification, is a rare subtype of MDS/MPN with aggressive clinical outcomes [1]. aCML is characterized by recurrent somatic mutations in *SETBP1*, *ASXL1*, and *ETNK1* genes, as well as high genetic heterogeneity [11,12], although their prognostic impact remains unclear. According to the fifth edition of the WHO classification, MDS/MPN-RS-T, now classified as MDS/MPN with *SF3B1* mutation and thrombocytosis (MDS/MPN-T-SF3B1), has the most favorable prognosis among all MDS/MPN types [1]. Previous studies indicate that frameshift and nonsense *ASXL1* mutations may be prognostic [6,13]. However, Mangaonkar et al.’s study, which includes 158 MDS/MPN-RS-T patients, did not identify molecular abnormalities as predictors of survival in MDS/MPN-RS-T [14]. Finally, MDS/MPN-U has been renamed as MDS/MPN-not otherwise specified (MDS/MPN NOS), according to the fifth edition of the WHO classification. Several molecular signatures have been identified in MDS/MPN-U, with overlap between CMML, aCML, and MDS/MPN-RS-T [1]. Therefore, a better understanding of the molecular features will aid in comprehending disease evolution, diagnosis, and prognosis, and has the potential for the development of targeted therapies.

The purpose of this study is to investigate the genetic characteristics of different MDS/MPN subtypes using comprehensive next-generation sequencing (NGS) panels, with the aim of identifying potential diagnostic and prognostic molecular signatures that could be applied in clinical practice.

## 2. Results

### 2.1. Case Cohort Characteristics

**Figure 1:**
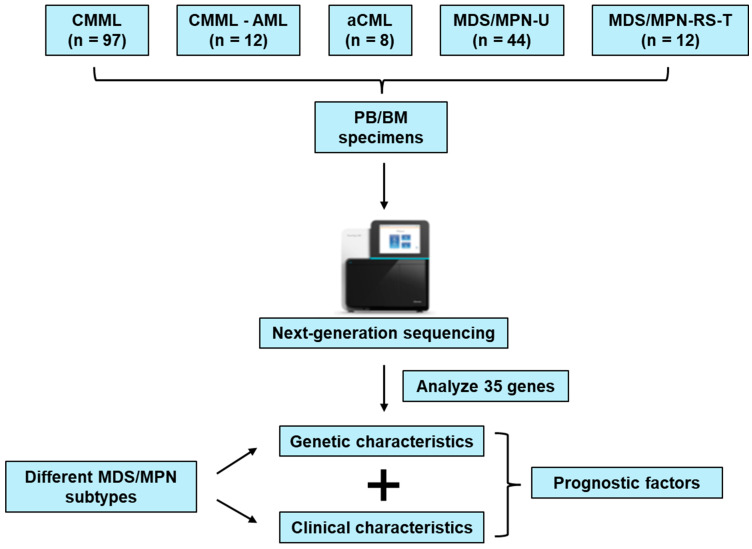
The workflow and study design of our cohort. BM, bone marrow; PB, peripheral blood. (Abbreviations: aCML, atypical myeloid leukemia; AML, acute myeloid leukemia; CMML, chronic myelomonocytic leukemia; MDS/MPN-U, myelodysplastic/myeloproliferative neoplasm-unclassified; and MDS/MPN-RS-T, myelodysplastic/myeloproliferative neoplasm with ring sideroblasts and thrombocytosis.)

Our study included 173 patients, comprising CMML (n = 97), CMML-AML (n = 12), aCML (n = 8), MDS/MPN-U (n = 44), and MDS/MPN-RS-T (n = 12). The mean age of the patients was 68.3 years (range: 34–89 years), with 120 males (69.4%) and 53 females (30.6%). An abnormal karyotype was observed most frequently in CMML-AML (6/12, 50%) and aCML (4/8, 50%), followed by MDS/MPN-U (20/44, 45.5%), CMML (33/97, 34.0%), and MDS/MPN-RS-T (4/12, 33.3%). Complex karyotypes (≥3 alterations) were identified in 26.6% of patients (46/173), most commonly in CMML-AML (5/12, 41.7%) and MDS/MPN-U (15/44, 34.1%). These findings are consistent with previous studies [6,15]. The clinical and pathological features of these patients are summarized in Table 1, and the workflow is shown in Figure 1.

### 2.2. Overall Molecular Signatures in MDS/MPN

**Figure 2:**
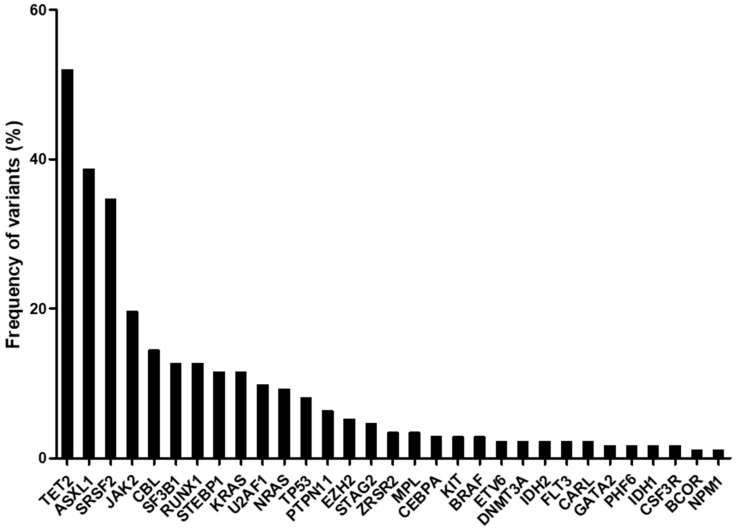
Frequency of recurrent gene mutations in all myelodysplastic/myeloproliferative neoplasm (MDS/MPN) patients (n = 173).

A mean of 3.1 pathogenic/likely pathogenic variants per patient (range: 0 to 11; median: 3) was identified among the different MDS/MPN subtypes. Analyzing mutation frequencies within the total cohort revealed that *TET2* (52.0%) was the most frequently mutated gene, followed by *ASXL1* (38.7%), *SRSF2* (34.7%), *JAK2* (19.7%), *CBL* (14.5%), *SF3B1* (12.7%), *RUNX1* (12.7%), *SETBP1* (11.6%), and *KRAS* (11.6%). All other investigated genes showed mutation frequencies <10% (Figure 2). Similar findings were reported in other MDS/MPN studies [6,15,16].

### 2.3. Molecular Signatures among Different MDS/MPN Subtypes

**Figure 3:**
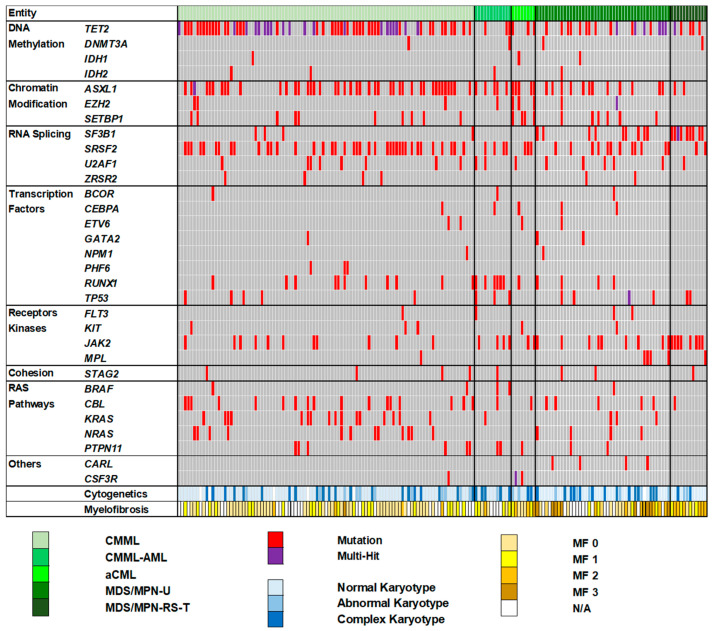
Molecular and cytogenetic characteristics among the different MDS/MPN subtypes (n = 173). An oncoplot showing the mutated genes among the different MDS/MPN subtypes. Each column represents a patient. Thirty-one genes are grouped into eight categories based on their functions: DNA methylation, chromatin modification, RNA splicing, transcription factors, receptor kinases, cohesion, RAS pathways, and others. Green depicts the different MDS/MPN subtypes: CMML, CMML-AML, aCML, MDS/MPN-U, and MDS/MPN-RS-T. Red depicts a single gene mutation; purple depicts more than one mutation in the same gene, mainly corresponding to biallelic *TET2* mutations. Cytogenetic findings are divided into three groups: normal karyotype, abnormal karyotype, and complex karyotype. Myelofibrosis (MF) status is divided into five groups: MF 0, MF 1, MF 2, MF 3, and N/A. The frequency of recurrent gene mutations among the different MDS/MPN subtypes. (Abbreviations: aCML, atypical myeloid leukemia; AML, acute myeloid leukemia; CMML, chronic myelomonocytic leukemia; MDS/MPN-U, myelodysplastic/myeloproliferative neoplasm-unclassified; MDS/MPN-RS-T, myelodysplastic/myeloproliferative neoplasm with ring sideroblasts and thrombocytosis; MF, myelofibrosis; and N/A, not applicable.)

The recurrent gene mutations and cytogenetic characteristics among the different MDS/MPN subtypes are summarized in Figure 3. Interestingly, we observed that the distribution of mutation frequencies differed across the various MDS/MPN subtypes.

**Figure 4:**
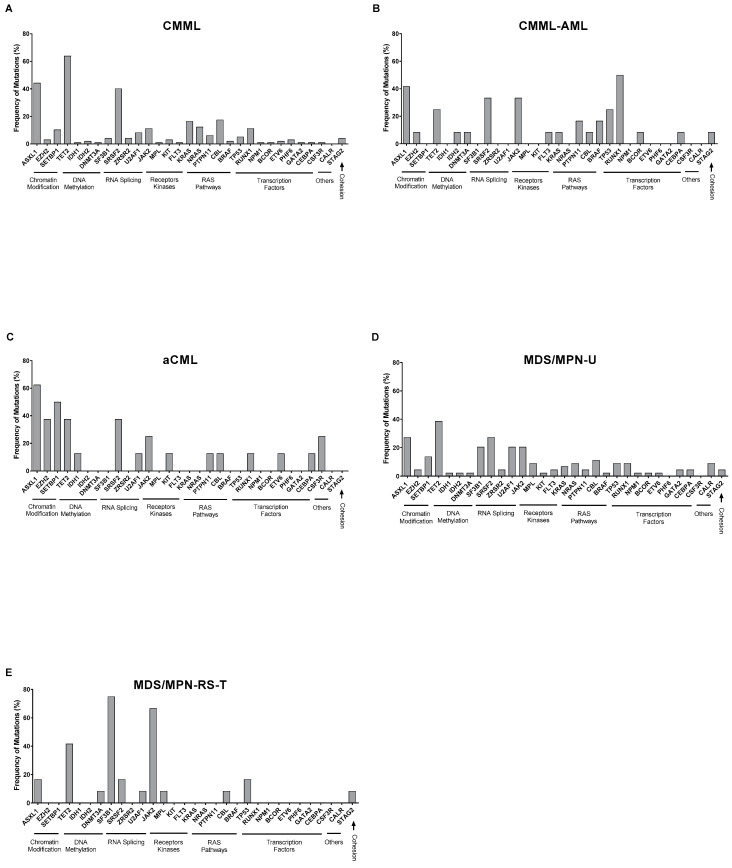
Frequency of mutations based on functional classification among different MDS/MPN subtypes. (**A**) CMML (n = 97); (**B**) CMML-AML (n = 12); (**C**) aCML (n = 8); (**D**) MDS/MPN-U (n = 44); and (**E**) MDS/MPN-RS-T (n = 12). (Abbreviations: aCML, atypical myeloid leukemia; AML, acute myeloid leukemia; CMML, chronic myelomonocytic leukemia; MDS/MPN-U, myelodysplastic/myeloproliferative neoplasm-unclassified; and MDS/MPN-RS-T, myelodysplastic/myeloproliferative neoplasm with ring sideroblasts and thrombocytosis).

As illustrated in Figure 4A, *TET2* (62/97; 63.9%), *ASXL1* (43/97; 44.3%), and *SRSF2* (39/97; 40.2%) were the most common recurrent mutations in CMML patients involving DNA methylation, chromatin modification, and RNA splicing pathways. These findings are consistent with previous studies [6,7]. Notably, *TET2* multi-hit mutations (indicated by the purple color in Figure 3) were particularly common in CMML (20/62; 32.3%) compared to other MDS/MPN subtypes. Additionally, *TET2* mutations were commonly associated with *SRSF2* (35/97; 36.1%). We also observed that CMML-AML patients showed a high frequency of *RUNX1* mutations (6/12; 50%) compared to CMML (11/97; 11.3%) patients, indicating that the *RUNX1* mutation may have been acquired later during AML transformation (Figure 4B). We found that all patients who underwent AML transformation were originally diagnosed with CMML.

Regarding aCML, these cases were characterized by frequent mutations in *ASXL1* (5/8; 62.5%), *SETBP1* (4/8; 50%), *EZH2* (3/8; 37.5%), and *TET2* (3/8; 37.5%) (Figure 4C). *SETBP1* mutations have been described as a marker associated with the diagnosis of aCML, which aligns with our findings here [11,17].

Compared to other MDS/MPN subtypes, MDS/MPN-U patients exhibited the most heterogeneous mutational profiles, with high frequencies observed in *TET2* (17/44; 38.6%), *ASXL1* (12/44; 27.3%), *SRSF2* (12/44, 27.3%), *JAK2* (9/44; 20.5%), and *SF3B1* (9/44; 20.5%) (Figure 4D). Most mutations occurred at known “hotspots”, which is consistent with previous studies [16].

In contrast, MDS/MPN-RS-T patients demonstrated much less heterogeneity, with the majority of recurrent mutations involving *SF3B1* (9/12; 75%), *JAK2* (8/12; 66.7%), and *TET2* (5/12; 41.7%). Interestingly, we noticed that, except for one patient with a *CBL* mutation, no mutations related to RAS pathways (*BRAF*, *KRAS*, *NRAS*, and *PTPN11*) were identified in these MDS/MPN-RS-T patients (Figure 4E).

To gain further insights into the different mechanisms related to various MDS/MPN subtypes, recurrent mutation genes were analyzed based on their functional classification (Supplementary Tables S1 and S2 and Supplementary Figure S1). As shown in Supplementary Figure S1, the group of chromatin modification-related genes was more often mutated in aCML (7/8; 87.5%), while the group of RNA splicing-associated genes was most often mutated in MDS/MPN-RS-T (12/12; 100%). Mutations in receptor kinase genes occurred at a lower frequency in CMML (15/97; 15.5%) compared to MDS/MPN-RS-T (9/12; 75%) and CMML-AML (5/12; 41.7%). In contrast, RAS pathway genes were less common in MDS/MPN-RS-T (1/12; 8.3%) compared to CMML (46/97, 46.4%) and aCML (4/12; 33.3%). Genes related to transcription factors were most often mutated in CMML-AML (7/12; 58.3%).

### 2.4. Prognostic Analysis

The median OS of the entire cohort was 547 days (range: 14–6563 days). Twelve patients developed disease transformation to AML, with a median OS of 509 days (range: 67–2256 days) (Supplementary Figure S2). In the next step, univariate and multivariate analyses were performed to identify significant prognostic factors for OS in patients with CMML and MDS/MPN-U. The univariate analysis showed that abnormal karyotype and genetic abnormalities, including *NRAS*, *RUNX1*, *SETBP1,* and *TP53,* were associated with worse OS in CMML patients. However, in the multivariate analysis, older age (≥65 years), *RUNX1* mutation, and *TP53* mutation were independently correlated with an unfavorable clinical outcome in CMML (Table 2). For MDS/MPN-U, only the CBL mutation was identified as a worse prognosticator in both univariate and multivariate analyses (Table 3). Univariate and multivariate analyses were not performed for CMML-AML, aCML, and MDS/MPN-RS-T cases due to limited sample sizes.

## 3. Discussion

In this study, we utilized comprehensive NGS panels to characterize the genomic landscapes in patients with different MDS/MPN subtypes. Although our study indicated that no single gene mutation is specific to a particular MDS/MPN subtype, certain mutational signatures, in the context of appropriate clinical and morphological features, might be helpful in the diagnosis and prognosis of patients with MDS/MPN.

Our results demonstrated that MDS/MPN is characterized by the presence of mutations commonly identified in myeloid neoplasms, such as *TET2*, *ASXL1*, *SRSF2,* and *SF3B1*. However, the recurrence of these gene mutations varies among the MDS/MPN subtypes. For instance, we found that CMML showed a high frequency of *TET2* mutations (63.9%), which were commonly associated with a combination of biallelic *TET2* (32.3%) mutations [7,18]. Previous studies have shown that CMML clonal driver mutations can be detected in over 90% of cases, with the combination of *TET2* (particularly biallelic mutations) and *SRSF2* being highly specific for a myelomonocytic phenotype [19]. Conversely, we also noted that leukemia-associated driver mutations, including *NPM1* and *FLT3*, were very uncommon in CMML, as reported by Vallapureddy et al. [20]. Thus, these specific mutational signatures can provide supportive evidence for the diagnosis of CMML. MDS/MPN-RS-T is a unique entity characterized by a high frequency of *SF3B1* (75%) and *JAK2* (66.7%) mutations, as indicated in our study. The *SF3B1* mutation correlated strongly with ring sideroblasts in the bone marrow, and the presence of *SF3B1/JAK2* mutations along with ring sideroblasts and thrombocytosis can be used to establish the diagnosis of MDS/MPN-RS-T [1]. Furthermore, we found that aCML and MDS/MPN-U did not exhibit specific molecular features. aCML was characterized by high frequencies of *ASXL1* (62.5%) and *SETBP1* (50%) mutations, while MDS/MPN-U cases showed the most heterogeneous molecular features with mutations in *TET2* (38.6%), *ASXL1* (27.3%), *SRSF2* (27.3%), *JAK2* (20.5%), and *SF3B1* (20.5%). Although the presence of mutations in *SETBP1* supports the diagnosis of aCML, the overall mutation profile is similar to that of chronic neutrophilic leukemia, CMML, and MDS/MPN-U, as described previously [1,21].

Prognostic factors in different MDS/MPN subtypes have been investigated; however, the findings differ depending on the studies. Several studies have demonstrated that mutations in *RUNX1*, *NRAS*, *SETBP1*, and *ASXL1* are independently associated with a poor prognosis in CMML, while *TET2* mutations have been related to favorable outcomes [8,22,23,24]. We confirmed the negative prognostic effects of *RUNX1*, *NRAS*, and *SETBP1* mutations in CMML patients through univariate analysis, but we did not find any prognostic significance for *ASXL1* and *TET2* mutations. Furthermore, we identified an association between karyotype abnormalities and *TP53* mutations, with poorer outcomes in CMML patients; however, these associations were only significant for *RUNX1* and *TP53* in multivariate analysis. We believe that this discrepancy could be due to the limited sample size in our cohort. Compared to CMML, the prognostic factors in MDS/MPN-U have not been widely investigated. Recently, Mangaonkar et al. reported that *CBL* and *TP53* mutations are associated with poor prognosis, while *ASXL1* mutations were not predictive of OS [25]. In our analysis, we found that *CBL* mutations were linked to reduced OS in both univariate and multivariate analyses. However, we did not observe a prognostic impact of *TP53* mutations, likely due to the small number of patients with *TP53* mutations in our cohort. Regarding MDS/MPN-RS-T, the prognostic factors are controversial. Studies have suggested poor survival associated with the presence of *SETBP1* and *ASXL1* mutations [13]. However, a recent study by Mangaonkar et al., which included 158 MDS/MPN-RS-T cases, did not identify molecular abnormalities as predictors of survival in these patients [14]. We did not identify any mutations related to the OS; however, only 12 MDS/MPN-RS-T patients were included in our cohort.

Currently, allogeneic HSCT remains the only potentially curative option for patients with MDS/MPN, with hypomethylating agents (HMAs) serving as the mainstay of first-line chemotherapy for patients with MDS/MPN [26]. *ASXL1* and/or *NRAS* mutations have been identified as factors that may impact allogeneic HSCT outcomes in CMML [10]. A study by Karantanos et al. indicated that the presence of *SETBP1*, *RUNX1,* or *EZH2* mutations is associated with a worse response to HMAs [27]. Additionally, Duchmann et al. found that *ASXL1* mutations predicted a lower overall response rate to HMAs, while *TET2*^MT^/*ASXL1*^WT^ was associated with better OS in multivariate analysis [28]. Moreover, targetable therapies, such as RAS pathway inhibitors, have been shown to be effective in patients with CMML [29,30]. Therefore, gene mutations affecting prognosis may aid in clinical decision-making regarding the timing and necessity of allogeneic HSCT.

Our study has some limitations. Firstly, it is a retrospective study with heterogenous clinical and genomic information collected from three different medical centers. Secondly, while our cohort consists of 173 MDS/MPN patients, the sample size for aCML and MDS/MPN-RS-T is limited compared to other studies focused solely on these two entities [14,31]. Lastly, paired normal tissue with germline information was not available for our cases.

## 4. Materials and Methods

### 4.1. Patients and Specimens

This study was approved by the City of Hope Comprehensive Cancer Center Review Board (IRB #15198). A total of 173 patients diagnosed with MDS/MPN between January 1997 and April 2021, and treated at City of Hope (COH), University of Los Angeles Medical Center, or Dartmouth Hitchcock Medical Center, were included in our study. All of these patients met the diagnostic criteria outlined in the 2017 WHO Classification of Tumors of Haematopoietic and Lymphoid Tissues (WHO) for MDS/MPN [2]. The demographic, pathological, and molecular features of these patients were collected through chart reviews.

### 4.2. Next-Generation Sequencing Panels

The NGS panels from the aforementioned three medical centers, which included 54 to 340 cancer-associated genes, were used in our study. For consistency in comparison, we analyzed only 35 genes. No gene rearrangements were identified in our study. These NGS panels detect single-nucleotide variants (SNVs), short insertions/deletions, and copy number variants (CNVs). Peripheral blood or bone marrow specimens were the inputs for these NGS panels. The workflow included the acoustic shearing of isolated genomic DNA, library preparation, and library enrichment for specific genes of interest using a capture-based method. Pooled libraries were then sequenced on an Illumina sequencing instrument (San Diego, CA, USA). In general, if a variant is a frameshift, nonsense, or splice-site mutation of a tumor suppressor gene, it is classified as “pathogenic” or “likely pathogenic”. Missense or in-frame insertion/deletion mutations in mutational hotspots and critical domains of a well-studied protein are also classified as “pathogenic” or “likely pathogenic”.

### 4.3. Statistical Analysis

Baseline characteristics are presented as means and ranges for continuous variables and frequencies for categorical variables. OS is defined as the time from diagnosis to the last follow-up or death from any cause. The Cox proportional hazards regression model was used to identify significant factors for OS. A *p* value of < 0.05 was considered statistically significant. Data analyses were performed using either GraphPad Prism 5 software or IBM SPSS Statistics (Version 29).

## 5. Conclusions

Our study characterizes the mutational profiles among different MDS/MPN subtypes, potentially enhancing the diagnostic workup of MDS/MPN. Additionally, our findings confirm that specific gene mutations might be useful in evaluating the prognostic impact of different MDS/MPN subtypes, making them relevant for clinical decision-making. However, these findings need further validation in a larger sample size.

## Figures and Tables

**Table 1:** Demographic and clinical features of MD S/MPN patients (n = 173).

	All Cases (n = 173)	CMML (n = 97)	CMML-AML (n = 12)	aCML (n = 8)	MDS/MPN-U (n = 44)	MDS/MPN-RS-T (n = 12)
**Age (years); (mean, range)**	68.3 (34–89)	68.6 (34–87)	66.2 (40–74)	66.9 (49–85)	67.5 (34–89)	70.8 (53–87)
**Sex (n, %)**						
**Male**	120 (69.4%)	68 (70.1%)	9 (75%)	2 (25%)	34 (77.3%)	7 (58.3%)
**Female**	53 (30.6%)	29 (29.9%)	3 (25%)	6 (75%)	10 (22.7%)	5 (41.7%)
**Platelet (×10^9^/L)**	205.6 (11–1358)	147.4 (17–1296)	140.1 (30–524)	239.4 (61–542)	235.6 (11–1358)	702.1 (450–1290)
**Fibrosis (n, %)**						
**MF-0**	58 (33.5%)	44 (45.4%)	3 (25%)	1 (12.5%)	9 (20.5%)	1 (8.3%)
**MF-1**	32 (18.5%)	20 (20.6%)	4 (33.3%)	0	5 (11.4%)	3 (25%)
**MF-2**	16 (9.3%)	4 (4.1%)	1 (8.3%)	1 (12.5%)	10 (22.7%)	0
**MF-3**	12 (6.9%)	3 (1.0%)	0	1 (12.5%)	10 (22.7%)	0
**Unknown**	55 (31.8%)	28 (28.9%)	4 (33.4%)	5 (62.5%)	10 (22.7%)	8 (66.7%)
**Cytogenetics (n, %)**						
**Normal**	100 (57.8%)	59 (60.8%)	6 (50%)	4 (50%)	23 (52.2%)	8 (66.8%)
**Abnormal**	67 (38.7%)	33 (34.0%)	6 (50%)	4 (50%)	20 (45.5%)	4 (33.3%)
**Complex Karyotype (≥3)**	46 (26.6%)	22 (22.7%)	5 (41.7%)	2 (25%)	15 (34.1%)	2 (16.6%)
**Unknown**	6 (3.5%)	5 (5.2%)	0	0	1 (2.3%)	0
**Transplant (n, %)**						
**Yes**	41 (23.7%)	23 (23.7%)	3 (25%)	2 (25%)	12 (27.3%)	1 (8.3%)
**No**	130 (75.1%)	72 (74.2%)	9 (75%)	6 (75%)	32 (72.7%)	11 (91.7%)
**Unknown**	2 (1.2%)	2 (2.1%)	0	0	0	0
**Outcome**						
**Alive**	122 (70.5%)	71 (73.2%)	2 (16.7%)	7 (87.5%)	31 (70.5%)	11 (91.7%)
**Deceased**	51 (29.5%)	26 (26.8%)	10 (83.3%)	1 (12.5%)	13 (29.5%)	1 (8.3%)
**AML transformation (n, %)**	44 (25.4%)	26 (26.8%)	12 (100%)	1 (12.5%)	5 (11.4%)	0

Abbreviations: aCML, atypical myeloid leukemia; AML, acute myeloid leukemia; CMML, chronic myelomonocytic leukemia; MDS/MPN-U, myelodysplastic syndrome/myeloproliferative neoplasm-unclassified; MDS/MPN-RS-T, myelodysplastic syndrome/myeloproliferative neoplasm with ring sideroblasts and thrombocytosis; MF, Myelofibrosis.

**Table 2:** Univariate and multivariate analyses of clinical and genetic characteristics among patients with CMML.

Variable	Univariate (OS)		Multivariate (OS)	
	HR (95% CI)	*p* Value	HR (95% CI)	*p* Value
**Age (≥65 vs. <65)**	1.031 (0.354–3.006)	0.955	4.198(1.062–16.594)	0.041 *
**Gender (Female vs. Male)**	0.686 (0.275–1.714)	0.42	1.037 (0.373–2.884)	0.945
**Transplant (No vs. Yes)**	1.253 (0.497–3.158)	0.632	1.801 (0.644–5.036)	0.992
**Cytogenetics (Abnormal vs. Normal)**	2.549 (1.174–5.534)	0.018 *	1.715 (0.680–4.328)	0.253
***TET2*** ** (MT vs. WT)**	0.898 (0.396–2.035)	0.797	1.579 (0.434–5.751)	0.488
***ASXL1*** ** (MT vs. WT)**	1.895 (0.850–4.225)	0.118	0.951 (0.319–2.834)	0.927
***SRSF2*** ** (MT vs. WT)**	1.560 (0.711–3.427)	0.268	3.975 (1.139–13.869)	0.03
***CBL*** ** (MT vs. WT)**	0.616 (0.184–2.058)	0.431	0.280 (0.061–1.272)	0.099
***KRAS*** ** (MT vs. WT)**	0.695 (0.237–2.043)	0.507	0.484 (0.144–1.632)	0.242
***NRAS*** ** (MT vs. WT)**	2.537 (1.058–6.084)	0.037 *	8.269 (2.161–31.639)	0.002
***RUNX1*** ** (MT vs. WT)**	3.495 (1.438–8.493)	0.006 *	7.563 (2.305–24.807)	<0.001 *
***SETBP1*** ** (MT vs. WT)**	3.639 (1.355–9.773)	0.01 *	1.460 (0.330–6.460)	0.618
***U2AF1*** ** (MT vs. WT)**	1.026 (0.241–4.369)	0.972	5.529 (0.797–38.339)	0.083
***JAK2*** ** (MT vs. WT)**	0.513 (0.121–2.178)	0.365	0.225 (0.039–1.310)	0.097
***PTPN11*** ** (MT vs. WT)**	1.847 (0.434–7.852)	0.406	6.561 (0.837–51.400)	0.073
***TP53*** ** (MT vs. WT)**	3.696 (1.101–12.413)	0.034 *	20.524 (3.546–118.798)	<0.001 *

* Indicates statistical significance (*p* < 0.05) in predicting overall survival. Abbreviations: CI, confidence interval; CMML, chronic myelomonocytic leukemia; HR, hazard ratio; MT, mutant; OS, overall survival; WT, wild type.

**Table 3:** Univariate and multivariate analyses of clinical and genetic characteristics among patients with MDS/MNP-U.

Variable	Univariate (OS)		Multivariate (OS)	
	HR (95% CI)	*p* Value	HR (95% CI)	*p* Value
**Age (≥65 vs. <60)**	1.536 (0.418–5.649)	0.518	3.990 (0.544–29.243)	0.173
**Gender (Female vs. Male)**	0.279 (0.036–2.150)	0.22	0.471 (0.045–4.989)	0.532
**Transplant (No vs. Yes)**	2.991 (0.658–13.603)	0.156	19.039 (0.975–371.780)	0.052
**Cytogenetics (Abnormal vs. Normal)**	0.938 (0.314–2.801)	0.909	0.788 (0.191–3.249)	0.741
***TET2*** ** (MT vs. WT)**	1.733 (0.580–5.177)	0.325	2.802 (0.383–20.472)	0.31
***ASXL1*** ** (MT vs. WT)**	1.597 (0.522–4.885)	0.412	1.548 (0.251–9.528)	0.638
***SRSF2*** ** (MT vs. WT)**	0.787 (0.215–2.889)	0.719	0.229 (0.036–1.458)	0.119
***JAK2*** ** (MT vs. WT)**	1.062 (0.292–3.867)	0.927	0.916 (0.114–7.349)	0.934
***SF3B1*** ** (MT vs. WT)**	0.713 (0.157–3.228)	0.661	0.862 (0.089–8.306)	0.898
***SETBP1*** ** (MT vs. WT)**	1.191 (0.262–5.414)	0.821	1.369 (0.175–10.709)	0.765
***TP53*** ** (MT vs. WT)**	1.965 (0.434–8.887)	0.380	0.812 (0.095–6.924)	0.849
***CBL*** ** (MT vs. WT)**	3.286 (1.010–10.690)	0.048 *	25.796 (2.050–324.654)	0.012 *

* Indicates statistical significance (*p* < 0.05) in predicting overall survival. Abbreviations: CI, confidence interval; HR, hazard ratio; MDS/MPN-U, myelodysplastic/myeloproliferative neoplasm-unclassified; MT, mutant; OS, ovreall survival; WT, wild type.

## Data Availability

The data presented in this study are available on request from the corresponding author.

## Supplementary Materials

The following supporting information can be downloaded at: https://www.mdpi.com/article/10.3390/ijms251810214/s1.
