# Supplementary material for: Genomic Landscape of Myelodysplastic/Myeloproliferative Neoplasms: A Multi-Central Study
Source: Int J Mol Sci. 2024 Sep 23;25(18):10214. doi: 10.3390/ijms251810214 (PMC11431978; doi:10.3390/ijms251810214)
Supplement: Supplementary file 1 [file ijms-25-10214-s001.zip › ijms-3188326-supplementary.pdf]

Supplementary Table 1. Functional classification of genes in our cohort.

Functional Cluster	Genes							
RNA Splicing	*SF3B1*	*SRSF2*	*U2AF1*	*ZRSR2*				
Transcription Factors	*BCOR*	*CEBPA*	*ETV6*	*GATA2*	*NPM1*	*PHF6*	*RUNX1*	*TP53*
DNA Methylation	*DNMT3A*	*IDH1*	*IDH2*	*TET2*				
Chromatin Modification	*ASXL1*	*EZH2*	*SETBP1*					
Receptors/Kinases	*FLT3*	*KIT*	*JAK2*	*MPL*				
Cohesion	*STAG2*							
RAS Pathways	*BRAF*	*CBL*	*KRAS*	*NRAS*	*PTPN11*			
Others	*CARL*	*CSF3R*						

Supplementary Table 2. Frequency of mutations based on functional classification among different MDS/MPN subtypes.

Fucntional Classification	Frequency of Mutations (%)	Overall	CMML	CMML-AML	aCML	MDS/MPN-U	MDS/MPN-RS-T
**Chromatin Modification**	***ASXL1***	38.73	44.33	41.67	62.50	27.27	16.67
	EZH2	5.20	3.09	8.33	37.50	4.55	0.00
	SETBP1	11.56	10.31	0.00	50.00	13.64	0.00
**DNA Methylation**	***TET2***	52.02	63.92	25.00	37.50	38.64	41.67
	IDH1	1.73	1.03	0.00	12.50	2.27	0.00
	IDH2	2.31	2.06	8.33	0.00	2.27	0.00
	DNMT3A	2.31	1.03	8.33	0.00	2.27	8.33
**RNA Splicing**	***SF3B1***	12.72	4.12	0.00	0.00	20.45	75.00
	SRSF2	34.68	40.21	33.33	37.50	27.27	16.67
	ZRSR2	3.47	4.12	0.00	0.00	4.55	0.00
	U2AF1	9.83	8.25	0.00	12.50	20.45	8.33
**Receptors Kinases**	***JAK2***	19.65	11.34	33.33	25.00	20.45	66.67
	MPL	3.47	1.03	0.00	0.00	9.09	8.33
	KIT	2.89	3.09	0.00	12.50	2.27	0.00
	FLT3	2.31	1.03	8.33	0.00	4.55	0.00
**RAS Pathways**	***KRAS***	11.56	16.49	8.33	0.00	6.82	0.00
	NRAS	9.25	12.37	0.00	0.00	9.09	0.00
	PTPN11	6.36	6.19	16.67	12.50	4.55	0.00
	CBL	14.45	17.53	8.33	12.50	11.36	8.33
	BRAF	2.89	2.06	16.67	0.00	2.27	0.00
**Transcription Factors**	***TP53***	8.09	5.15	25.00	0.00	9.09	16.67
	RUNX1	12.72	11.34	50.00	12.50	9.09	0.00
	NPM1	1.16	1.03	0.00	0.00	2.27	0.00
	BCOR	1.16	1.03	8.33	0.00	2.27	0.00
	ETV6	2.31	2.06	0.00	12.50	2.27	0.00
	PHF6	1.73	3.09	0.00	0.00	0.00	0.00
	GATA2	1.73	1.03	0.00	0.00	4.55	0.00
	CEBPA	2.89	1.03	8.33	12.50	4.55	0.00
**Others**	***CSF3R***	1.73	1.03	0.00	25.00	0.00	0.00
	CALR	2.31	0.00	0.00	0.00	9.09	0.00
**Cohesion**	***STAG2***	4.62	4.12	8.33	0.00	4.55	8.33

Abbreviations: aCML, atypical myeloid leukemia; AML, acute myeloid leukemia; CMML, chronic myelomonocytic leukemia; MDS/MPN-U, myelodysplastic syndrome/myeloproliferative neoplasm-unclassified; MDS/MPN-RS-T, myelodysplastic syndrome/myeloproliferative neoplasm with ring sideroblasts and thrombocytosis; MF, Myelofibrosis.

Supplementary Figure 1. Frequency of recurrent mutations analyzed based on functional classification among different MDS/MPN subtypes (n = 107).

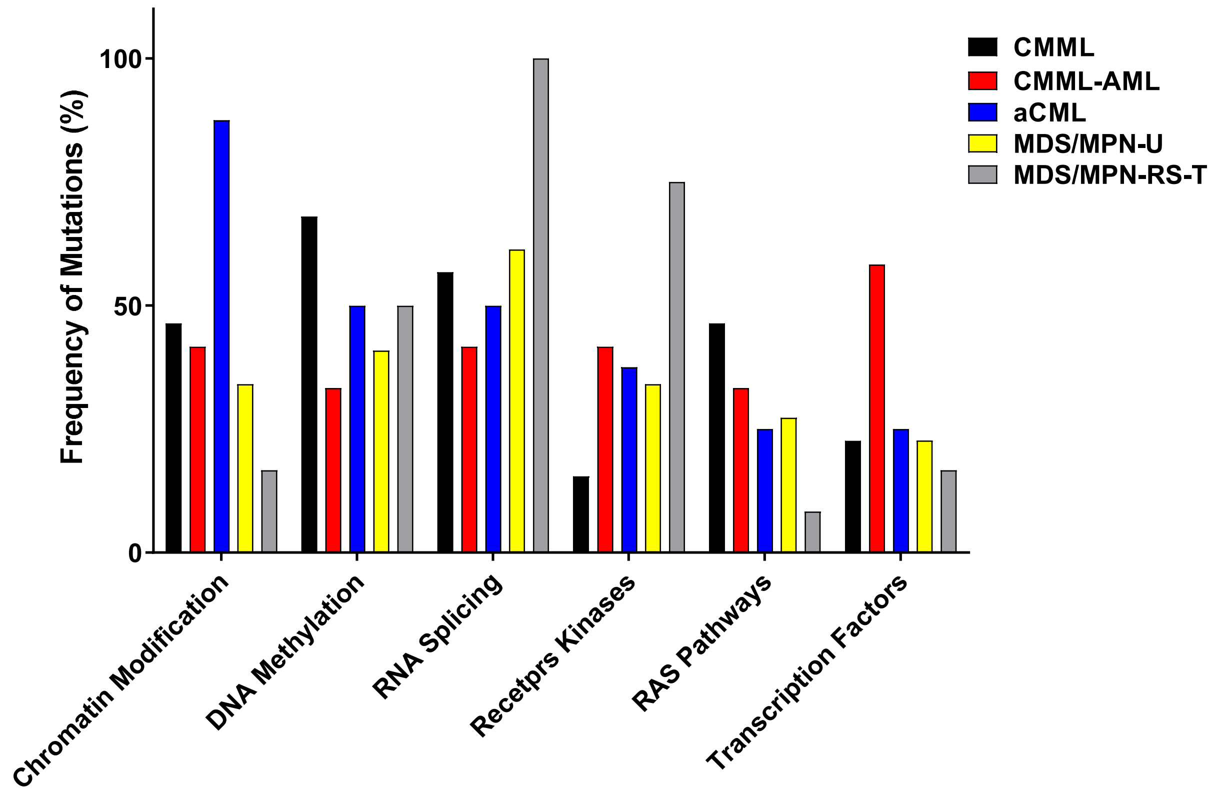

(Abbreviations: aCML, atypical myeloid leukemia; AML, acute myeloid leukemia; CMML, chronic myelomonocytic leukemia; MDS/MPN-U, myelodysplastic/myeloproliferative neoplasm-unclassified; MDS/MPN-RS-T, myelodysplastic/myeloproliferative neoplasm with ring sideroblasts and thrombocytosis.)

Supplementary Figure 2. Overall survival analysis among different MDS/MPN subtypes.

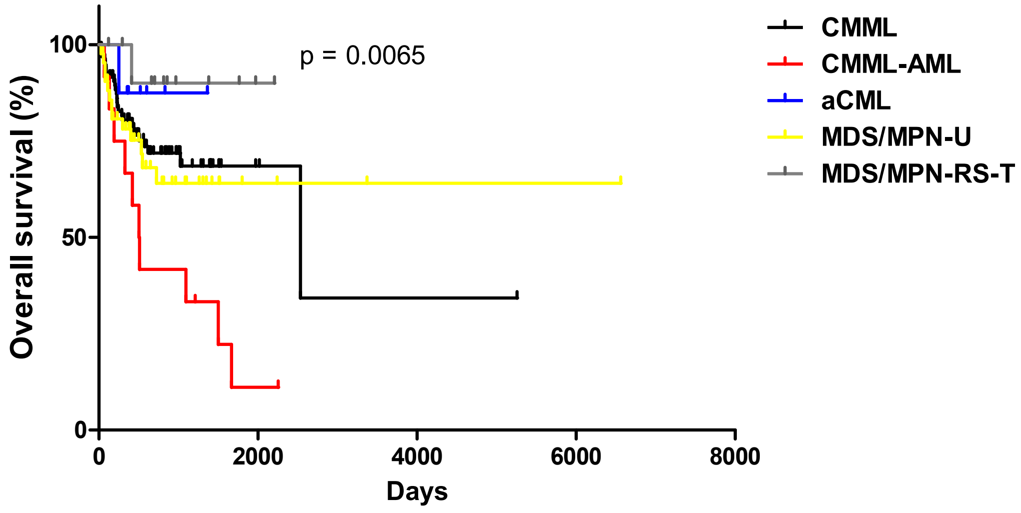

(Abbreviations: aCML, atypical myeloid leukemia; AML, acute myeloid leukemia; CMML, chronic myelomonocytic leukemia; MDS/MPN-U, myelodysplastic/myeloproliferative neoplasm-unclassified; MDS/MPN-RS-T, myelodysplastic/myeloproliferative neoplasm with ring sideroblasts and thrombocytosis.)
